# A life without worms

**DOI:** 10.1093/trstmh/trx010

**Published:** 2017-03-18

**Authors:** Richard E. Sanya, Gyaviira Nkurunungi, Irene Andia Biraro, Harriet Mpairwe, Alison M. Elliott

**Affiliations:** a MRC/UVRI Uganda Research Unit, Uganda Virus Research Institute, P.O. Box 49, Entebbe, Uganda; b College of Health Sciences, Makerere University, Kampala, Uganda; c Department of Clinical Research, London School of Hygiene & Tropical Medicine, Keppel Street, London WC1E 7HT, UK

**Keywords:** Allergy, Anthelminthic, Helminths, Infectious diseases, Metabolic disease, Vaccines

## Abstract

Worms have co-evolved with humans over millions of years. To survive, they manipulate host systems by modulating immune responses so that they cause (in the majority of hosts) relatively subtle harm. Anthelminthic treatment has been promoted as a measure for averting worm specific pathology and to mitigate subtle morbidities which may include effects on anaemia, growth, cognitive function and economic activity. With our changing environment marked by rapid population growth, urbanisation, better hygiene practices and anthelminthic treatment, there has been a decline in worm infections and other infectious diseases and a rise in non-communicable diseases such as allergy, diabetes and cardiovascular disease. This review reflects upon our age-old interaction with worms, and the broader ramifications of life without worms for vaccine responses and susceptibility to other infections, and for allergy-related and metabolic disease. We touch upon the controversy around the benefits of mass drug administration for the more-subtle morbidities that have been associated with worm infections and then focus our attention on broader, additional aspects of life without worms, which may be either beneficial or detrimental.

## Introduction

Over a billion people are estimated to be infected with helminths, most living in areas of poverty.^1,2^ Helminths have co-existed with mammals for millions of years. Their lifecycles have evolved to ensure their survival while minimising harm to the mammalian host. Soil transmitted helminths (STH) such as hookworm, *Ascaris lumbricoides, Trichuris trichiura* and *Strongyloides stercoralis* spend part of their lifecycle in soil and gain access to their human host through skin penetration or ingestion. For the filarial nematodes, an insect vector takes up microfilariae during a blood meal and, after development in the insect, the parasite is injected into the next host during another blood meal. For water borne helminths such as *Schistosoma*, cercariae shed by the snail intermediate host access the definitive human host by skin penetration during contact with infested water. After migratory and development stages, adult worms lodge in body tissues such as the gut, blood vessels and lymphatics. In most cases, helminths do not replicate in the mammalian host.^3^

Helminths induce short and long term morbidity, and pathology in some body systems: gastrointestinal tract (malabsorption, diarrhoea, macro and micronutrient deficiencies, bleeding, intestinal obstruction, rectal prolapse), liver (peri-portal fibrosis, cholangitis, cholangiocarcinoma, hepatocellular carcinoma), cardiovascular system (anaemia), lymphatic system (lymphoedema), central nervous system (blindness, epilepsy), genitourinary tract (haematuria, hydronephrosis, bladder cancer), lungs (Loeffler's syndrome).^4^ These effects depend on the type and number of helminths in the host. Treatment is deserved to avert these harmful effects.

Societies in developing countries are experiencing remarkable population growth, urbanisation and lifestyle changes. With better hygiene and ‘deworming’ interventions, helminth infections are declining. Concurrently, there is a rise in non-communicable diseases (NCDs) such as diabetes^5^ and cardiovascular diseases,^6^ contributing significantly to global mortality and attributed largely to changes in diet and lifestyle. Could the decline in helminth infections be playing a role in this epidemiological transition? With helminth elimination the ultimate goal of mass drug administration (MDA) programmes,^7^ it is of interest to reflect on the prospect of a worm-free life. Do we clearly understand, are we ready for, the consequences of life divorced from the partnership established over millions of years? In this narrative review we discuss current evidence regarding the benefits of MDA, ways in which worms manipulate us, and the possible effects of helminth infection on responses to vaccines and unrelated infectious diseases, and on allergy and metabolic disease.

## How much do we benefit from MDA?

MDA entails administration of anthelminthic medicines without reference to an individual's infection status, or test of cure. The World Health Assembly endorsed MDA for school children as a schistosomiasis and STH control strategy for high transmission settings^8^ and this has been widely adopted.

MDA policy is premised on anticipated benefits for helminth-specific pathology, maternal anaemia, birth weight, childhood growth, anaemia, cognitive function, school performance and long term economic returns. We do not question the benefit of MDA for controlling pathologies such as schistosome-induced fibrosis, hookworm-induced anaemia, elephantiasis and river blindness. However, the impact of MDA on more subtle morbidities associated with helminths has been difficult to demonstrate.

Mass treatment for hookworm in the American south at the turn of 20th century was associated with greater school enrolment, attendance and literacy and long-term gain in income.^9^ Further, in 2004, Miguel and Kremmer published a highly influential report showing an association between school-based MDA and reduced school absenteeism among Kenyan children.^10^ Ten years later, these children who were dewormed at school had more years of school enrolment, more time in employment and longer work hours each week.^11^ However, recent reanalyses of the original data have highlighted the challenges of evaluating such interventions.^12,13^

A large cluster-randomised trial in India with one million pre-school children showed little effect of regular deworming on mortality in pre-school children.^14^ A Cochrane review^15^ concluded that treating children known to have STH may improve weight gain but evidence of benefits on haemoglobin, school attendance and cognitive function is limited; also that community based treatment programmes had little or no effect on these outcomes. Similarly, a systematic review found the evidence insufficient to link helminths to cognitive performance^16^ and a further meta-analysis concluded that mass deworming of children had little or no effect on weight, height, cognition, school attendance or mortality.^17^

WHO recommends anthelminthic treatment during pregnancy, hoping that it will reduce maternal anaemia, increase birth weight and reduce mortality. The benefits are not yet clear.^18^ We, and others, have found limited overall effects^19^ of anthelminthic use during pregnancy on maternal anaemia, and none on birth weight, perinatal mortality or congenital abnormalities.^20–23^ Anthelminthic treatment during pregnancy did not affect infectious disease incidence or response to immunisation.^24,25^ A Cochrane review notes that evidence is insufficient to recommend use of anthelminthic medication for pregnant women in the first trimester and administration of a single dose anthelminthic was not associated with any impact on maternal anaemia.^26^

There has been debate on the policy of MDA^27^ and systematic review methodology has been questioned in its application to helminths.^28,29^ However, it brings to light the need for more evidence to support MDA and to understand fully its benefits.

## How do worms manipulate us?

**Figure 1.**
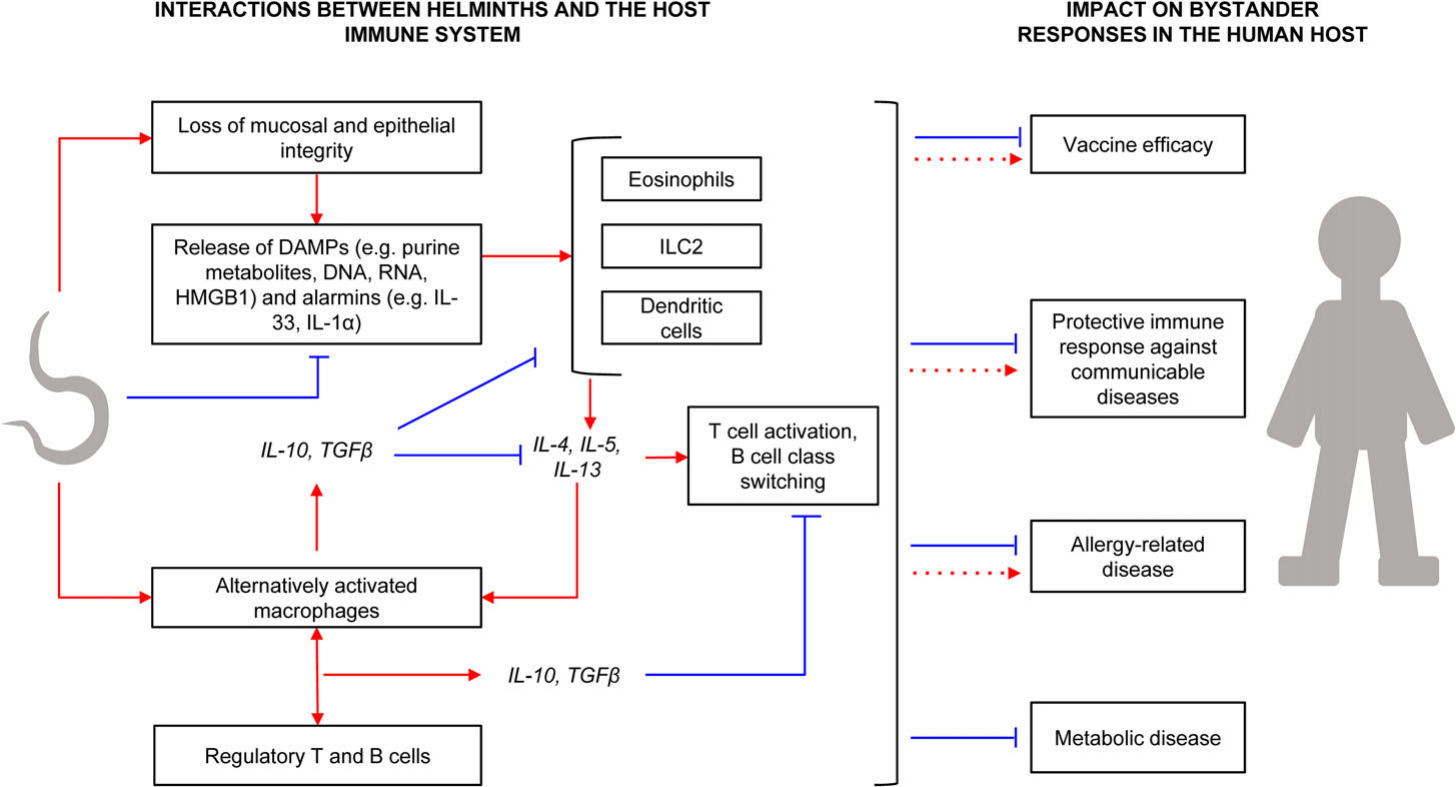
Interactions between helminths and the host immune system, and the impact on bystander responses. Red arrows and blue lines denote positive and suppressive effects, respectively. Helminth migration in the host results in tissue injury, resulting in release of Damage Associated Molecular Patterns (DAMPs) and alarmins. DAMPs and alarmins are involved in the initial activation of eosinophils, type 2 innate lymphoid cells (ILC2) and antigen presenting cells (APCs) such as dendritic cells (DCs), which then mediate further inflammation in the host. However, some helminth secretory products can suppress alarmin release and DC maturation, and some helminth enzymes degrade DAMPs. Helminths also interfere with APC activities, promoting an alternative activation phenotype, which results in production of large amounts of IL-10 and TGFβ. These cytokines downmodulate eosinophil, ILC2 and DC responses, and promote lymphocyte hyporesponsiveness involving regulatory lymphocytes. Helminth interaction with host immunity has spillover effects on responses to bystander antigens. For instance, helminth infections may result in impaired immune responses to vaccines and communicable diseases, although specific helminth molecules may actually have enhancing effects. Likewise, there is evidence for both inverse and positive helminth-allergy associations, although any notable effects on metabolic conditions have been beneficial. DNA: Deoxyribonucleic acid; RNA: Ribonucleic acid; HMGB1: High Mobility Group Box 1.

The age-old colonisation of mammals by helminths has been successful mainly because of the latter's shrewd manipulation of host systems (Figure 1).

Helminths employ enzymes and other excretory/secretory proteins to disrupt and alter host tissues, thus successfully migrating, feeding, establishing niches and developing strategies to exit the host to complete their life-cycles. For example, *Schistosoma* cercariae contain proteases that aid in skin penetration^30^ while the major excretory/secretory protein of *Trichuris trichiura* induces pore formation to facilitate helminth entrenchment in the gut.^31^

Loss of mucosal and epithelial integrity as helminths take root in the host is often accompanied by release of inflammatory mediators, such as alarmins and damage associated molecular patterns,^32^ normally detrimental to the helminth and its host. However, helminth excretory/secretory products also work to offset this. For example, secreted products of *Heligmosomoides polygyrus* block production of the alarmin IL-33, a key inducer of Th2-type inflammation.^33^ Besides, to curb host morbidity from helminth-inflicted tissue injury, helminth-induced mediators spearhead tissue healing and remodelling.^34^

But perhaps the helminth's most potent survival weapon is the wide array of mechanisms developed to evade or regulate a vigilant host immune system. At the helm are helminth-induced Th2-type and regulatory immune responses. Helminth-induced Th2 cytokines interleukin (IL)-4 and IL-13 promote alternative activation of macrophages, resulting in production of large amounts of immunomodulatory IL-10 and transforming growth factor (TGF)-β,^35^ and T cell hypo-responsiveness involving regulatory T cells.^36^ Helminth-induced, IL-10-producing ‘regulatory B cells’ have also been demonstrated.^37^ A notable consequence of helminth-induced immunomodulation is the attenuation of responses to ‘bystander’ antigens,^38^ widely implicated in the helminth-associated modulation of immune responses to a number of non-communicable and communicable diseases.

Helminth-allergy interactions are a good example of the bystander effect. Although antigenic targets for allergen- and helminth-specific immune responses are similar,^39^ helminth infections seem to be protective against allergy-related conditions in both humans and mice. Current evidence^32,40^ points to an extensive array of immunomodulatory mechanisms underlying inverse helminth-allergy associations. They range from induction of IL-10-producing regulatory T cells, regulatory B cells and alternatively activated antigen presenting cells, promotion of polyclonal IgE synthesis and immunoglobulin class switching to IgG4, to suppression of release of alarmins (such as IL-33) and inhibition of type 2 innate lymphoid cell activity.

Host metabolic responses may also be influenced by helminth infections. For example, *S. mansoni* egg antigen-treated obese mice have increased levels of white adipose tissue Th2-type cells, modified macrophage activation and reduced adipose tissue mass and improved insulin sensitivity.^41^ Non-obese diabetic mice infected with *H. polygyrus* and *Trichinella spiralis* are protected against type-1 diabetes through the Th2-associated reduction of inflammatory autoimmune responses.^42^ There is also recent evidence in humans and mice that helminths may protect against inflammatory bowel diseases through Th2-type immunity-mediated expansion of a protective microbiota.^43^

Helminth-induced bystander response suppression is a double-edged sword. We may benefit from helminth-driven regulation of non-communicable diseases, as elaborated above. However, helminth excretory/secretory products and Th2 cytokines have been shown to suppress anti-microbial functions of innate immune cells (dendritic cells and macrophages), leading to increased differentiation of regulatory T cells and Th2 cells while impeding the development of protective Th1-type responses and potentially compromising immunity to several communicable diseases.

## Worms and vaccines

Following recognition of the Th1/Th2 hypothesis,^44^ the contrasting ability of mycobacterial and helminth antigens to elicit Th1 and Th2 responses, respectively, and mutual inhibition between these opposing effects,^45^ it was proposed that helminth co-infection might account for the poor efficacy of vaccines such as BCG in tropical settings and the high prevalence of TB and HIV in Africa.^46^ As helminth prevalence declines, will vaccines become more effective, and susceptibility to other infectious diseases decrease?

Studies in animal models largely suggest that this will be the case. In the mouse, infection with *H. polygyrus* (a nematode with a life-cycle confined to the gut) modified the response to a malaria protein vaccine, resulting in reduced antibody and Th1 responses, increased Th2 and regulatory responses and impaired protection against malaria challenge.^47^ Treatment of the helminth before, but not after, immunisation abrogated these effects, emphasising the importance of co-infection at the time of immunisation. Similar effects of *H. polygyrus* have been reported for a DNA malaria vaccine, but not for live, irradiated sporozoites,^48^ or for live BCG,^49^ indicating that the impact of a particular helminth differs by vaccine type: protein, DNA or live attenuated organisms. Mice infected with *Schistosoma* species (which cause systemic infections) show impaired induction of protective immunity both to malaria^50^ and to TB challenge (following BCG),^51^ indicating that different helminth infections have different effects. *Schistosoma* infections also resulted in impaired antibody responses to toxoid and protein vaccines—but a study on hepatitis B immunisation showed a gradual recovery of the response when the infection was treated after immunisation.^52^ The life-cycle of *Trichinella spiralis* involves an intestinal phase, followed by encystment in skeletal muscle; suppression of the IgA response to cholera toxin^53^ and to hepatitis B immunisation^54^ has been demonstrated during the intestinal, but not the muscle, stages of the life-cycle. While these experiments demonstrate suppressive effects, intraperitoneal injection of *Ascaris* extract concurrently with BCG has been shown to enhance macrophage activation and suppress BCG replication^55^ and a protein from the filarial worm *Onchocerca volvulus* shows promise as an adjuvant for influenza vaccine.^56^ Together, studies in mice show that helminth infections have important potential to supress vaccine responses, but that helminth species, stage of life-cycle, timing of helminth exposure and treatment, and characteristics of the vaccine may be important determinants of the outcome and that specific helminth molecules may actually have enhancing effects. As well, differences between murine models are likely to result from genetics of the host and intensity of helminth infection used.

In humans, studies of the impact of helminth co-infection on vaccine responses are important in their own right, and also offer an important surrogate for studies on susceptibility to infections, which are much more difficult to undertake. The bystander modulatory effects of chronic helminth infections are of potential direct significance in adolescents and adults when primary or recall immunisation occurs in this age group. For example, for human papilloma virus immunisation, tetanus and other boosters, and during outbreaks, such as the recent Yellow Fever and Ebola epidemics; also when novel vaccines are undergoing initial evaluations in older populations. Observational studies among children and adults have shown associations between helminth infection and suppression of antibody and Th1 responses, particularly during systemic filarial infections and schistosomiasis: vaccines affected include BCG, tetanus, typhoid and a candidate malaria vaccine.^57–65^ Hepatitis B immunisation may also be impaired in the context of schistosomiasis^66^ but effects may be limited to those with hepatosplenic disease, calling into question the causal mechanisms involved.^67^ Clinical trials may help us to test whether helminth induced immunomodulation is causal in suppression of vaccine responses and, so far, these have been confined to effects of geohelminths. Treatment of geohelminths with albendazole has been shown to improve the Th1 response to BCG,^68,69^ and the antibody and Th1 response to oral cholera vaccine.^70,71^ No studies have yet investigated the effects of treating schistosomiasis or filariases but, on balance, the data so far suggest that vaccine responses will improve with the elimination of worms.

However, the majority of vaccines in current use target pathogens that cause substantial disease and death in early life. They are administered to the very youngest age groups in whom chronic helminth infections have yet to establish themselves. In these age groups, it is maternal infection status that is potentially of greatest importance in terms of impacting on a newborn's capacity for induction of vaccine-specific responses. Evidence that the human fetus could be sensitised in utero to helminths and mycobacterial antigen^72^ suggested that prenatal exposure might influence infant vaccine responses. Indeed, initial studies by Malhotra and colleagues showed an association between sensitisation to *Schistosoma* or filarial antigens in utero and a Th2 bias to the infant response to BCG immunisation.^73^ Malhotra and colleagues also described adverse associations between prenatal exposure to hookworm and other helminths and the response to diphtheria toxoid and *Haemophilus influenzae* type B (HiB) immunisation in infancy,^74^ but this has not been confirmed by results from Uganda where the only association observed was a possible enhancement of IgG responses to pertussis toxin, HiB and hepatitis B among infants of mothers with *Strongyloides*.^25,75,76^ A study in Ecuador also showed no association between exposure to maternal geohelminths and infant responses to diphtheria toxoid, tetanus, pertussis, measles, Rubella or HiB, but enhanced IgA responses to polio and rotavirus.^77^ An important consideration is that the infant outcome may vary depending on the nature and timing of the exposure to parasite antigens: Malhotra and colleagues showed that infant DT responses were enhanced if the infant was sensitised to malaria antigens, but suppressed if the infant was ‘tolerised’.^74^ Only one substantive trial has investigated the effects of treating helminths during pregnancy on infant vaccine responses: this did not confirm findings from an earlier pilot^78^ and gave only weak evidence of an effect of treating maternal hookworm on the infant response to tetanus or BCG immunisation.^24,25^ Further work is needed to understand whether helminth elimination among pregnant women will alter the infant response to key vaccines.

Given the complex effects of helminths on vaccine responses it is not surprising that effects on infectious disease susceptibility are complex too (reviewed elsewhere).^79–81^ A possible unifying hypothesis, supported by recent evidence from mouse models,^82^ is that chronic helminth co-infection has little effect on the innate response to incident infections (and may even enhance it) but does impair adaptive responses that control replication of established infections. For example, in the case of TB, a recent trial on effects of anthelminthic treatment on bovine TB among wild buffalo in South Africa's Kruger National Park found that regular anthelminthic treatment had no impact on *Mycobacterium bovis* infection incidence, but resulted in lower mortality among *M. bovis* infected animals.^83^ Similarly, we found little evidence that helminth co-infection affects susceptibility to TB infection in humans,^84^ but recent results suggest that treatment of helminths may abrogate regulatory T cells-mediated suppression of Th1 cell frequency and function in helminth-TB co-infection^85^ and hints at improved clinical outcome.^86^

## Worms and allergy-related disease

Results from epidemiological studies on the relationship between helminths and allergy have been inconsistent. As for vaccine studies, different helminth species interact with the host's immune system differently, resulting in different clinical outcomes. An earlier review and meta-analysis^87^ found that hookworm had an inverse association with asthma (summary odds ratio [OR] 0.50, 95% CI 0.28–0.90), with a ‘dose-response’ by infection intensity, *A. lumbricoides* showed a positive association and *T. trichiura* showed no relationship. Another meta-analysis^88^ showed an inverse association between helminthic infections and allergen skin sensitisation (summary OR 0.69, 95% CI 0.6–0.79).

Exposure to helminth infections in-utero and in early childhood is negatively associated with allergy risk in childhood. Our birth cohort in Uganda showed that maternal hookworm during pregnancy was associated with a reduced incidence of eczema in childhood (adjusted hazard ration [aHR] 0.71, 95% CI 0.51–0.99), with a dose-response, and that early childhood infections with *T. trichiura* and hookworm were associated with a reduced incidence of childhood eczema.^89^ Treatment of maternal helminths during pregnancy increased the incidence of eczema in childhood.^24,90^ A study in Brazil also showed that early childhood infections with *T. trichiura* and *A. lumbricoides* were associated with a lower prevalence of allergen skin reactivity in later childhood.^91^ In Gabon, a lower prevalence of skin reactivity to house dust mite was reported among children infected with *Schistosoma haematobium* compared to those without the infection.^92^ Most studies have considered helminths as an independent variable in regression models, but there is increasing evidence that helminths are effect-modifiers of the relationship between atopy and clinical allergy. We found that maternal hookworm during pregnancy attenuated the association between *Dermatophagoides*-specific IgE and eczema in childhood, as well as the effects of other known risk-factors for eczema such as mother's history of eczema and female gender.^89^ This effect-modification has also been reported in studies in Ecuador.^93, 94^ A study conducted in Uganda found a positive association between *Dermatophagoides*-specific IgE and histamine release among children without hookworm but not amongst children with hookworm.^95^

Despite the inconsistencies outlined, epidemiological studies have consistently shown a lower prevalence of clinical allergy (and sometimes atopy) in rural compared to urban areas in low and middle income countries.^96–98^ This is consistent with the observed low prevalence of asthma/allergy among children raised on farms compared to city dwellers in high income countries.^97^ In the high income countries, this farm effect has been attributed to exposure to diverse microbiome on the farm and to the consumption of unpasteurised dairy products.^98^ For low and middle income countries, the protective effect had been attributed partly to geohelminths,^93^ but the possible role of the microbiome has not yet been extensively explored. Animal studies have demonstrated interactions between helminths and microbiota.^99^ Could the microbiome in rural settings explain why, in Ugandan island communities, we found a very low prevalence of clinical allergies, despite positive associations between helminths and reported wheeze (and atopy)?^100^

Additionally, there is increasing evidence of attenuation of the relationship between atopy and allergy among children in rural compared to urban areas in low and middle income countries.^93,101,102^ This has been attributed partly to geohelminths, but the role of other infections and microbiome deserves investigation.

Studies on immigrants from rural to urban setting represent natural experiments. One such study^103^ found that immigrants from rural Ethiopia to Israel had a low prevalence of atopy and allergy, and a negative association between helminth infection and atopy on arrival, which was quickly reversed after a year of living in Israel. This was attributed to the treatment of helminths, a decline in helminths among the untreated, and exposure to a novel environment.

The helminth-allergy relationship is complicated by the many inter-related factors at play. To obtain a conclusive stand, we need to conduct comprehensive studies that take into account the various helminth-related variables, and the potential interaction and confounding with the microbiome, other infections (such as malaria) and interaction and other environmental exposures. This will require extensive data collection and advanced statistical analyses. But the potential benefits are worth it, for we will be able to understand better how to harness the beneficial effects of worms or the rural environment for the primary, secondary and tertiary prevention of asthma, allergies and other chronic inflammatory conditions that may be associated with a life without worms.

## Worms and metabolic disease

A recent systematic review showed that individuals with a previous or current helminth infection were 50% less likely to have metabolic dysfunction.^104^

In diet induced obese mice, chronic infection with *Schistosoma mansoni* lowered whole body insulin resistance and glucose intolerance and improved peripheral glucose uptake and insulin sensitivity. Injection of schistosome antigens induced a similar effect^41^ and, in a separate study, reduced atherosclerosis in mice.^105^ Mice infected with *H. polygyrus* had lower blood glucose, insulin resistance, fat accumulation than uninfected mice^106^ and benefits were sustained even after clearance of the helminth.^107^*Nippostrongylus brasiliensis* infection was associated with decreased weight gain and improved glucose metabolism.^108^ Similarly, diet induced obese mice infected with *Litomosoides sigmodontis* or exposed to its antigen had improved glucose tolerance.^109^

In humans, a cross-sectional study in rural China showed that individuals with a history of schistosomiasis infection exhibited lower fasting blood glucose levels compared to controls who had never had schistosomiasis.^110^ A study in India^111^ reported a lower prevalence of filarial infections in patients with type 2 diabetes than in non-diabetic controls. Patients with type 2 diabetes and lymphatic filariasis had lower concentrations of pro-inflammatory cytokines—IL-6 and GM-CSF—than patients without lymphatic filariasis. Among aboriginal adults in Australia, prior *Strongyloides stercoralis* infection was associated with reduced type 2 diabetes risk.^112^ Infection with STH has also been associated with decreased insulin resistance and lower body mass index, abdominal obesity, and lipid levels.^113,114^

Together, these recent findings indicate that helminth infections may convey important benefits for metabolic disease in humans. If so, understanding the mechanisms with a view to harnessing this knowledge for prevention and therapy of metabolic disease is important.

## Conclusions

**Figure 2.**
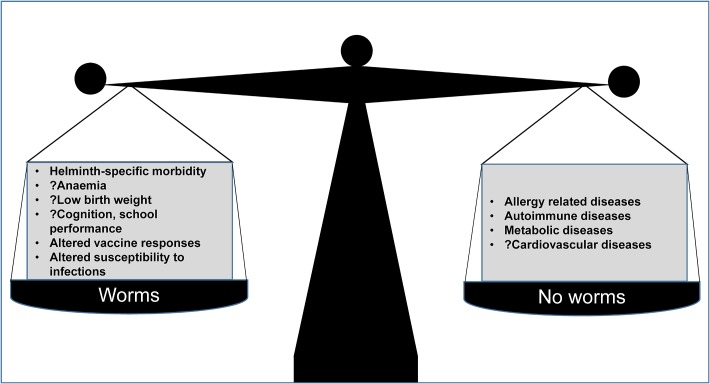
Is ‘de-worming’ good for us?

Helminths can be damaging, especially when there are intense infections: therefore, control is good. Some authors have also argued that MDA is a cost-effective health investment for governments^115^ although, as we have discussed, controlled trials to date have struggled to confirm a major impact of MDA on the subtle morbidities and mortality associated with worm infections in observational studies. As the debate on MDA continues, we need to note that removal of helminths leaves the immune system out of balance. We postulate that helminth elimination will result in a broad array of additional effects, both beneficial and detrimental to human health. The consequences may include altered responses to vaccines and to infectious diseases, and increased susceptibility to inflammatory conditions such as allergy-related disease and metabolic disease (Figure 2). Further work is needed to understand helminth-human interactions and their mechanisms, so that we can mitigate adverse consequences in the event that helminth infections in humans are eliminated.

## References

[1] Pullan RL, Smith JL, Jasrasaria R et al Global numbers of infection and disease burden of soil transmitted helminth infections in 2010. Parasit Vectors 2014;7:37. 24447578 10.1186/1756-3305-7-37 PMC3905661

[2] Colley DG, Bustinduy AL, Secor WE et al Human schistosomiasis. Lancet 2014;383:2253–64. 24698483 10.1016/S0140-6736(13)61949-2 PMC4672382

[3] Hotez PJ, Brindley PJ, Bethony JM et al Helminth infections: the great neglected tropical diseases. J Clin Invest 2008;118:1311–21. 18382743 10.1172/JCI34261 PMC2276811

[4] Farrar J, Hotez P, Junghanss T et al Manson's Tropical Diseases. 23rd ed Amsterdam: Elsevier Saunders; 2013.

[5] Danaei G, Finucane MM, Lu Y et al National, regional, and global trends in fasting plasma glucose and diabetes prevalence since 1980: systematic analysis of health examination surveys and epidemiological studies with 370 country-years and 2.7 million participants. Lancet 2011;378:31–40. 21705069 10.1016/S0140-6736(11)60679-X

[6] Danaei G, Finucane MM, Lin JK et al National, regional, and global trends in systolic blood pressure since 1980: systematic analysis of health examination surveys and epidemiological studies with 786 country-years and 5.4 million participants. Lancet 2011;377:568–77. 21295844 10.1016/S0140-6736(10)62036-3

[7] WHO Accelerating work to overcome the global impact of neglected tropical diseases. A roadmap to implemention. Geneva: World Health Organization; 2012 http://www.who.int/neglected_diseases/NTD_RoadMap_2012_Fullversion.pdf [accessed 26 December 2016].

[8] WHO The Fifty-fourth World Health Assembly. Agenda item 13.3. Schistosomiasis and soil-transmitted helminth infections. Geneva: World Health Organization; 2001 http://apps.who.int/gb/archive/pdf_files/WHA54/ea54r19.pdf [accessed 27 December 2016].

[9] Bleakley H Disease and development: evidence from hookworm eradication in the American South. Q J Econ 2007;122:73–117. 24146438 10.1162/qjec.121.1.73 PMC3800113

[10] Miguel E, Kremer M; Worms: Identifying impacts on education and health in the presence of treatment externalities. Econometrica 2004;72:159–217.

[11] Baird S, Hicks JH, Kremer M et al Worms at work: long-run impacts of a child health investment. Q J Econ 2016;131:1637–80. 27818531 10.1093/qje/qjw022 PMC5094294

[12] Aiken AM, Davey C, Hargreaves JR et al Re-analysis of health and educational impacts of a school-based deworming programme in western Kenya: a pure replication. Int J Epidemiol 2015;44:1572–80. 26203169 10.1093/ije/dyv127 PMC4681107

[13] Davey C, Aiken AM, Hayes RJ et al Re-analysis of health and educational impacts of a school-based deworming programme in western Kenya: a statistical replication of a cluster quasi-randomized stepped-wedge trial. Int J Epidemiol 2015;44:1581–92. 26203171 10.1093/ije/dyv128 PMC4681108

[14] Awasthi S, Peto R, Read S et al Population deworming every 6 months with albendazole in 1 million pre-school children in North India: DEVTA, a cluster-randomised trial. Lancet 2013;381:1478–86. 23498850 10.1016/S0140-6736(12)62126-6 PMC3647147

[15] Taylor-Robinson DC, Maayan N, Soares-Weiser K et al Deworming drugs for soil-transmitted intestinal worms in children: effects on nutritional indicators, haemoglobin, and school performance. Cochrane Database Syst Rev 2015:1–157. CD000371. 10.1002/14651858.CD000371.pub6 PMC4523932 26202783

[16] Dickson R, Awasthi S, Williamson P et al Effects of treatment for intestinal helminth infection on growth and cognitive performance in children: systematic review of randomised trials. BMJ 2000;320:1697–701. 10864543 10.1136/bmj.320.7251.1697 PMC27412

[17] Welch VA, Ghogomu E, Hossain A et al Mass deworming to improve developmental health and wellbeing of children in low-income and middle-income countries: a systematic review and network meta-analysis. Lancet Glob Health 2017;5:e40–e50. 27955788 10.1016/S2214-109X(16)30242-X

[18] Mpairwe H, Tweyongyere R, Elliott A Pregnancy and helminth infections. Parasite Immunol 2014;36:328–37. 24471654 10.1111/pim.12101 PMC4260141

[19] Torlesse H, Hodges M Albendazole therapy and reduced decline in haemoglobin concentration during pregnancy (Sierra Leone). Trans R Soc Trop Med Hyg 2001;95:195–201. 11355560 10.1016/s0035-9203(01)90164-6

[20] Ndibazza J, Muhangi L, Akishule D et al Effects of deworming during pregnancy on maternal and perinatal outcomes in Entebbe, Uganda: a randomized controlled trial. Clin Infect Dis 2010;50:531–40. 20067426 10.1086/649924 PMC2857962

[21] Larocque R, Casapia M, Gotuzzo E et al A double-blind randomized controlled trial of antenatal mebendazole to reduce low birthweight in a hookworm-endemic area of Peru. Trop Med Int Health 2006;11:1485–95. 17002722 10.1111/j.1365-3156.2006.01706.x

[22] Gyorkos TW, Larocque R, Casapia M et al Lack of risk of adverse birth outcomes after deworming in pregnant women. Pediatr Infect Dis J 2006;25:791–4. 16940835 10.1097/01.inf.0000234068.25760.97

[23] Olveda RM, Acosta LP, Tallo V et al Efficacy and safety of praziquantel for the treatment of human schistosomiasis during pregnancy: a phase 2, randomised, double-blind, placebo-controlled trial. Lancet Infect Dis 2016;16:199–208. 26511959 10.1016/S1473-3099(15)00345-X PMC4752899

[24] Webb EL, Mawa PA, Ndibazza J et al Effect of single-dose anthelmintic treatment during pregnancy on an infant's response to immunisation and on susceptibility to infectious diseases in infancy: a randomised, double-blind, placebo-controlled trial. Lancet 2011;377:52–62. 21176950 10.1016/S0140-6736(10)61457-2 PMC3018567

[25] Nash S, Mentzer AJ, Lule SA et al The impact of prenatal exposure to parasitic infections and to anthelminthic treatment on antibody responses to routine immunisations given in infancy: secondary analysis of a randomised controlled trial. PLoS Negl Trop Dis 2017;11:e0005213. 28178298 10.1371/journal.pntd.0005213 PMC5298230

[26] Salam RA, Haider BA, Humayun Q et al Effect of administration of antihelminthics for soil-transmitted helminths during pregnancy. Cochrane Database Syst Rev 2015:1–32. CD005547. 10.1002/14651858.CD005547.pub3 26087057

[27] Allen T, Parker M Deworming delusions? Mass drug administration in East African schools. J Biosoc Sci 2016;48(Suppl 1):S116–47. 27428063 10.1017/S0021932016000171

[28] Bundy DA, Kremer M, Bleakley H et al Deworming and development: asking the right questions, asking the questions right. PLoS Negl Trop Dis 2009;3:e362. 19172186 10.1371/journal.pntd.0000362 PMC2627944

[29] Campbell SJ, Nery SV, Doi SA et al Complexities and perplexities: a critical appraisal of the evidence for soil-transmitted helminth infection-related morbidity. PLoS Negl Trop Dis 2016;10:e0004566. 27196100 10.1371/journal.pntd.0004566 PMC4873196

[30] Ingram J, Knudsen G, Lim KC et al Proteomic analysis of human skin treated with larval schistosome peptidases reveals distinct invasion strategies among species of blood flukes. PLoS Negl Trop Dis 2011;5:e1337. 21980548 10.1371/journal.pntd.0001337 PMC3181243

[31] Drake L, Korchev Y, Bashford L et al The major secreted product of the whipworm, Trichuris, is a pore-forming protein. Proc Biol Sci 1994;257:255–61. 7991635 10.1098/rspb.1994.0123

[32] Maizels RM, McSorley HJ, Smyth DJ Helminths in the hygiene hypothesis: sooner or later. Clin Exp Immunol 2014;177:38–46. 24749722 10.1111/cei.12353 PMC4089153

[33] McSorley HJ, Blair NF, Smith KA et al Blockade of IL-33 release and suppression of type 2 innate lymphoid cell responses by helminth secreted products in airway allergy. Mucosal Immunol 2014;7:1068–78. 24496315 10.1038/mi.2013.123 PMC4016792

[34] Allen JE, Sutherland TE Host protective roles of type 2 immunity: parasite killing and tissue repair, flip sides of the same coin. Semin Immunol 2014;26:329–40. 25028340 10.1016/j.smim.2014.06.003 PMC4179909

[35] Mosser DM, Edwards JP Exploring the full spectrum of macrophage activation. Nat Rev Immunol 2008;8:958–69. 19029990 10.1038/nri2448 PMC2724991

[36] Taylor MD, van der Werf N, Maizels RM T cells in helminth infection: the regulators and the regulated. Trends Immunol 2012;33:181–9. 22398370 10.1016/j.it.2012.01.001

[37] Mangan NE, Fallon RE, Smith P et al Helminth infection protects mice from anaphylaxis via IL-10-producing B cells. J Immunol 2004;173:6346–56. 15528374 10.4049/jimmunol.173.10.6346

[38] Wammes LJ, Hamid F, Wiria AE et al Regulatory T cells in human geohelminth infection suppress immune responses to BCG and *Plasmodium falciparum*. Eur J Immunol 2010;40:437–42. 20063313 10.1002/eji.200939699

[39] Tyagi N, Farnell EJ, Fitzsimmons CM et al Comparisons of allergenic and Metazoan parasite proteins: allergy the price of immunity. PLoS Comput Biol 2015;11(10):e1004546. 26513360 10.1371/journal.pcbi.1004546 PMC4626114

[40] Hamid F, Amoah AS, van Ree R et al Helminth-induced IgE and protection against allergic disorders. Curr Top Microbiol Immunol 2015;388:91–108. 25553796 10.1007/978-3-319-13725-4_5

[41] Hussaarts L, Garcia-Tardon N, van Beek L et al Chronic helminth infection and helminth-derived egg antigens promote adipose tissue M2 macrophages and improve insulin sensitivity in obese mice. FASEB J 2015;29:3027–39. 25852044 10.1096/fj.14-266239

[42] Saunders KA, Raine T, Cooke A et al Inhibition of autoimmune type 1 diabetes by gastrointestinal helminth infection. Infect Immun 2007;75:397–407. 17043101 10.1128/IAI.00664-06 PMC1828378

[43] Ramanan D, Bowcutt R, Lee SC et al Helminth infection promotes colonization resistance via type 2 immunity. Science 2016;352:608–12. 27080105 10.1126/science.aaf3229 PMC4905769

[44] Mosmann TR, Cherwinski H, Bond MW et al Two types of murine helper T cell clone. I. Definition according to profiles of lymphokine activities and secreted proteins. J Immunol 1986;136:2348–57. 2419430

[45] Del Prete GF, De Carli M, Mastromauro C et al Purified protein derivative of *Mycobacterium tuberculosis* and excretory-secretory antigen(s) of Toxocara canis expand in vitro human T cells with stable and opposite (type 1 T helper or type 2 T helper) profile of cytokine production. J Clin Invest 1991;88:346–50. 1829097 10.1172/JCI115300 PMC296040

[46] Bentwich Z, Kalinkovich A, Weisman Z et al Can eradication of helminthic infections change the face of AIDS and tuberculosis. Immunol Today 1999;20:485–7. 10529774 10.1016/s0167-5699(99)01499-1

[47] Su Z, Segura M, Stevenson MM Reduced protective efficacy of a blood-stage malaria vaccine by concurrent nematode infection. Infect Immun 2006;74:2138–44. 16552043 10.1128/IAI.74.4.2138-2144.2006 PMC1418908

[48] Noland GS, Chowdhury DR, Urban JF Jr et al Helminth infection impairs the immunogenicity of a *Plasmodium falciparum* DNA vaccine, but not irradiated sporozoites, in mice. Vaccine 2010;28:2917–23. 20188676 10.1016/j.vaccine.2010.02.055 PMC2846978

[49] Rafi W, Bhatt K, Gause WC et al Neither primary nor memory immunity to *Mycobacterium tuberculosis* infection is compromised in mice with chronic enteric helminth infection. Infect Immun 2015;83:1217–23. 25605766 10.1128/IAI.03004-14 PMC4333454

[50] Laranjeiras RF, Brant LC, Lima AC et al Reduced protective effect of *Plasmodium berghei* immunization by concurrent *Schistosoma mansoni* infection. Mem Inst Oswaldo Cruz 2008;103:674–7. 19057817 10.1590/s0074-02762008000700008

[51] Elias D, Akuffo H, Pawlowski A et al *Schistosoma mansoni* infection reduces the protective efficacy of BCG vaccination against virulent *Mycobacterium tuberculosis*. Vaccine 2005;23:1326–34. 15661380 10.1016/j.vaccine.2004.09.038

[52] Chen L, Liu WQ, Lei JH et al Chronic *Schistosoma japonicum* infection reduces immune response to vaccine against hepatitis B in mice. PLoS One 2012;7:e51512. 23272112 10.1371/journal.pone.0051512 PMC3522692

[53] Ljungstrom I, Holmgren J, Huldt G et al Effect of experimental trichinosis on intestinal secretion and on local antibody formation to cholera toxin. Scand J Infect Dis Suppl 1980;(Suppl 24):79–81. 6937981

[54] Guan F, Hou X, Nie G et al Effect of *Trichinella spiralis* infection on the immune response to HBV vaccine in a mouse model. Foodborne Pathog Dis 2013;10:882–7. 23883369 10.1089/fpd.2013.1545

[55] Ferreira AP, Aarestrup FM, Bonecini-Almeida MG et al Effect of the injection of an extract of *Ascaris suum* on macrophage activation during the early phase of *Mycobacterium bovis* BCG infection in C57Bl/6 mice. Braz J Med Biol Res 1999;32:1429–36. 10559845 10.1590/S0100-879X1999001100014

[56] Jiang J, Fisher EM, Hensley SE et al Antigen sparing and enhanced protection using a novel rOv-ASP-1 adjuvant in aqueous formulation with influenza vaccines. Vaccine 2014;32:2696–702. 24681229 10.1016/j.vaccine.2014.03.046 PMC4080630

[57] Kilian HD, Nielsen G Cell-mediated and humoral immune response to tetanus vaccinations in onchocerciasis patients. Trop Med Parasitol 1989;40:285–91. 2617034

[58] Kilian HD, Nielsen G Cell-mediated and humoral immune responses to BCG and rubella vaccinations and to recall antigens in onchocerciasis patients. Trop Med Parasitol 1989;40:445–53. 2623427

[59] Sabin EA, Araujo MI, Carvalho EM et al Impairment of tetanus toxoid-specific Th1-like immune responses in humans infected with *Schistosoma mansoni*. J Infect Dis 1996;173:269–72. 8537675 10.1093/infdis/173.1.269

[60] Nookala S, Srinivasan S, Kaliraj P et al Impairment of tetanus-specific cellular and humoral responses following tetanus vaccination in human lymphatic filariasis. Infect Immun 2004;72:2598–604. 15102768 10.1128/IAI.72.5.2598-2604.2004 PMC387878

[61] Prost A, Schlumberger M, Fayet MT Response to tetanus immunization in onchocerciasis patients. Ann Trop Med Parasitol 1983;77:83–5. 6882059 10.1080/00034983.1983.11811675

[62] Grove DI, Forbes IJ Immunosuppression in bancroftian filariasis. Trans R Soc Trop Med Hyg 1979;73:23–6. 375486 10.1016/0035-9203(79)90123-8

[63] Cooper PJ, Espinel I, Paredes W et al Impaired tetanus-specific cellular and humoral responses following tetanus vaccination in human onchocerciasis: a possible role for interleukin-10. J Infect Dis 1998;178:1133–8. 9806045 10.1086/515661

[64] Muniz-Junqueira MI, Tavares-Neto J, Prata A et al Antibody response to *Salmonella typhi* in human schistosomiasis mansoni. Rev Soc Bras Med Trop 1996;29:441–5. 8885672 10.1590/s0037-86821996000500006

[65] Esen M, Mordmuller B, de Salazar PM et al Reduced antibody responses against *Plasmodium falciparum* vaccine candidate antigens in the presence of *Trichuris trichiura*. Vaccine 2012;30:7621–4. 23085365 10.1016/j.vaccine.2012.10.026

[66] Ghaffar YA, Kamel M, Abdel Wahab MF et al Hepatitis B vaccination in children infected with *Schistosoma mansoni*: correlation with ultrasonographic data. Am J Trop Med Hyg 1990;43:516–9. 2146894 10.4269/ajtmh.1990.43.516

[67] Bassily S, Strickland GT, Abdel-Wahab MF et al Efficacy of hepatitis B vaccination in primary school children from a village endemic for *Schistosoma mansoni*. J Infect Dis 1992;166:265–8. 1386097 10.1093/infdis/166.2.265

[68] Elias D, Britton S, Aseffa A et al Poor immunogenicity of BCG in helminth infected population is associated with increased in vitro TGF-beta production. Vaccine 2008;26:3897–902. 18554755 10.1016/j.vaccine.2008.04.083

[69] Elias D, Wolday D, Akuffo H et al Effect of deworming on human T cell responses to mycobacterial antigens in helminth-exposed individuals before and after bacille Calmette-Guerin (BCG) vaccination. Clin Exp Immunol 2001;123:219–25. 11207651 10.1046/j.1365-2249.2001.01446.x PMC1905995

[70] Cooper PJ, Chico M, Sandoval C et al Human infection with *Ascaris lumbricoides* is associated with suppression of the interleukin-2 response to recombinant cholera toxin B subunit following vaccination with the live oral cholera vaccine CVD 103-HgR. Infect Immun 2001;69:1574–80. 11179329 10.1128/IAI.69.3.1574-1580.2001 PMC98058

[71] Cooper PJ, Chico ME, Losonsky G et al Albendazole treatment of children with ascariasis enhances the vibriocidal antibody response to the live attenuated oral cholera vaccine CVD 103-HgR. J Infect Dis 2000;182:1199–206. 10979918 10.1086/315837

[72] Malhotra I, Ouma J, Wamachi A et al In utero exposure to helminth and mycobacterial antigens generates cytokine responses similar to that observed in adults. J Clin Invest 1997;99:1759–66. 9120021 10.1172/JCI119340 PMC507997

[73] Malhotra I, Mungai P, Wamachi A et al Helminth- and Bacillus Calmette-Guerin-induced immunity in children sensitized in utero to filariasis and schistosomiasis. J Immunol 1999;162:6843–8. 10352306

[74] Malhotra I, McKibben M, Mungai P et al Effect of antenatal parasitic infections on anti-vaccine IgG levels in children: a prospective birth cohort study in Kenya. PLoS Negl Trop Dis 2015;9:e0003466. 25590337 10.1371/journal.pntd.0003466 PMC4295886

[75] Elliott AM, Mawa PA, Webb EL et al Effects of maternal and infant co-infections, and of maternal immunisation, on the infant response to BCG and tetanus immunisation. Vaccine 2010;29:247–55. 21040693 10.1016/j.vaccine.2010.10.047 PMC3021124

[76] Kizito D, Tweyongyere R, Namatovu A et al Factors affecting the infant antibody response to measles immunisation in Entebbe-Uganda. BMC Public Health 2013;13:619. 23816281 10.1186/1471-2458-13-619 PMC3733798

[77] Clark CE, Fay MP, Chico ME et al Maternal helminth infection is associated with higher infant immunoglobulin A titers to antigen in orally administered vaccines. J Infect Dis 2016;213:1996–2004. 26908751 10.1093/infdis/jiw066 PMC4878726

[78] Elliott AM, Namujju PB, Mawa PA et al A randomised controlled trial of the effects of albendazole in pregnancy on maternal responses to mycobacterial antigens and infant responses to Bacille Calmette-Guerin (BCG) immunisation [ISRCTN32849447]. BMC Infect Dis 2005;5:115. 16371154 10.1186/1471-2334-5-115 PMC1352364

[79] Degarege A, Veledar E, Degarege D et al *Plasmodium falciparum* and soil-transmitted helminth co-infections among children in sub-Saharan Africa: a systematic review and meta-analysis. Parasit Vectors 2016;9:344. 27306987 10.1186/s13071-016-1594-2 PMC4908807

[80] Salgame P, Yap GS, Gause WC Effect of helminth-induced immunity on infections with microbial pathogens. Nat Immunol 2013;14:1118–26. 24145791 10.1038/ni.2736 PMC4955540

[81] Means AR, Burns P, Sinclair D et al Antihelminthics in helminth-endemic areas: effects on HIV disease progression. Cochrane Database Syst Rev 2016;4:Cd006419. 27075622 10.1002/14651858.CD006419.pub4 PMC4963621

[82] Scheer S, Krempl C, Kallfass C et al *S. mansoni* bolsters anti-viral immunity in the murine respiratory tract. PLoS One 2014;9:e112469. 25398130 10.1371/journal.pone.0112469 PMC4232382

[83] Ezenwa VO, Jolles AE Epidemiology. Opposite effects of anthelmintic treatment on microbial infection at individual versus population scales. Science 2015;347:175–7. 25574023 10.1126/science.1261714

[84] Biraro IA, Egesa M, Toulza F et al Impact of co-infections and BCG immunisation on immune responses among household contacts of tuberculosis patients in a Ugandan cohort. PLoS One 2014;9:e111517. 25372043 10.1371/journal.pone.0111517 PMC4221037

[85] Toulza F, Tsang L, Ottenhoff TH et al Mycobacterium tuberculosis-specific CD4+ T-cell response is increased, and Treg cells decreased, in anthelmintic-treated patients with latent TB. Eur J Immunol 2016;46(3):752–61. 26638865 10.1002/eji.201545843

[86] Abate E, Elias D, Getachew A et al Effects of albendazole on the clinical outcome and immunological responses in helminth co-infected tuberculosis patients: a double blind randomised clinical trial. Int J Parasitol 2015;45:133–40. 25486494 10.1016/j.ijpara.2014.09.006

[87] Leonardi-Bee J, Pritchard D, Britton J Asthma and current intestinal parasite infection: systematic review and meta-analysis. Am J Respir Crit Care Med 2006;174:514–23. 16778161 10.1164/rccm.200603-331OC

[88] Feary J, Britton J, Leonardi-Bee J Atopy and current intestinal parasite infection: a systematic review and meta-analysis. Allergy 2011;66:569–78. 21087217 10.1111/j.1398-9995.2010.02512.x

[89] Mpairwe H, Ndibazza J, Webb EL et al Maternal hookworm modifies risk factors for childhood eczema: results from a birth cohort in Uganda. Pediatr Allergy Immunol 2014;25:481–8. 25171741 10.1111/pai.12251 PMC4312885

[90] Ndibazza J, Mpairwe H, Webb EL et al Impact of anthelminthic treatment in pregnancy and childhood on immunisations, infections and eczema in childhood: a randomised controlled trial. PLoS One 2012;7:e50325. 23236367 10.1371/journal.pone.0050325 PMC3517620

[91] Rodrigues LC, Newcombe PJ, Cunha SS et al Early infection with *Trichuris trichiura* and allergen skin test reactivity in later childhood. Clin Exp Allergy 2008;38:1769–77. 18547322 10.1111/j.1365-2222.2008.03027.x

[92] van den Biggelaar AH, van Ree R, Rodrigues LC et al Decreased atopy in children infected with *Schistosoma haematobium*: a role for parasite-induced interleukin-10. Lancet 2000;356:1723–7. 11095260 10.1016/S0140-6736(00)03206-2

[93] Endara P, Vaca M, Platts-Mills TA et al Effect of urban vs. rural residence on the association between atopy and wheeze in Latin America: findings from a case-control analysis. Clin Exp Allergy 2015;45:438–47. 25200287 10.1111/cea.12399 PMC4413357

[94] Moncayo AL, Vaca M, Oviedo G et al Effects of geohelminth infection and age on the associations between allergen-specific IgE, skin test reactivity and wheeze: a case-control study. Clin Exp Allergy 2013;43:60–72. 23278881 10.1111/cea.12040 PMC3563216

[95] Pinot de Moira A, Fitzsimmons CM, Jones FM et al Suppression of basophil histamine release and other IgE-dependent responses in childhood *Schistosoma mansoni*/hookworm coinfection. J Infect Dis 2014;210:1198–206. 24782451 10.1093/infdis/jiu234 PMC4176447

[96] Hamid F, Wiria AE, Wammes LJ et al Risk factors associated with the development of atopic sensitization in Indonesia. PLoS One 2013;8:e67064. 23840583 10.1371/journal.pone.0067064 PMC3686782

[97] Scrivener S, Yemaneberhan H, Zebenigus M et al Independent effects of intestinal parasite infection and domestic allergen exposure on risk of wheeze in Ethiopia: a nested case-control study. Lancet 2001;358:1493–9. 11705561 10.1016/S0140-6736(01)06579-5

[98] von Mutius E, Vercelli D Farm living: effects on childhood asthma and allergy. Nat Rev Immunol 2010;10:861–8. 21060319 10.1038/nri2871

[99] Zaiss MM, Rapin A, Lebon L et al The intestinal microbiota contributes to the ability of helminths to modulate allergic inflammation. Immunity 2015;43:998–1010. 26522986 10.1016/j.immuni.2015.09.012 PMC4658337

[100] Webb EL, Nampijja M, Kaweesa J et al Helminths are positively associated with atopy and wheeze in Ugandan fishing communities: results from a cross-sectional survey. Allergy 2016;71:1156–69 26918891 10.1111/all.12867 PMC4949563

[101] Hamid F, Wahyuni S, van Leeuwen A et al Allergic disorders and socio-economic status: a study of schoolchildren in an urban area of Makassar, Indonesia. Clin Exp Allergy 2015;45:1226–36. 25703181 10.1111/cea.12517

[102] Obeng BB, Amoah AS, Larbi IA et al Schistosome infection is negatively associated with mite atopy, but not wheeze and asthma in Ghanaian schoolchildren. Clin Exp Allergy 2014;44:965–75. 24641664 10.1111/cea.12307

[103] Stein M, Greenberg Z, Boaz M et al The role of helminth infection and environment in the development of allergy: a prospective study of newly-arrived Ethiopian immigrants in Israel. PLoS Negl Trop Dis 2016;10:e0004208. 26752538 10.1371/journal.pntd.0004208 PMC4709081

[104] Tracey EF, McDermott RA, McDonald MI Do worms protect against the metabolic syndrome? A systematic review and meta-analysis. Diabetes Res Clin Pract 2016;120:209–20. 27596058 10.1016/j.diabres.2016.08.014

[105] Wolfs IM, Stoger JL, Goossens P et al Reprogramming macrophages to an anti-inflammatory phenotype by helminth antigens reduces murine atherosclerosis. FASEB J 2014;28:288–99. 24043262 10.1096/fj.13-235911

[106] Morimoto M, Azuma N, Kadowaki H et al Regulation of type 2 diabetes by helminth-induced Th2 immune response. J Vet Med Sci 2017;78:1855–64. 27665994 10.1292/jvms.16-0183 PMC5240765

[107] Wu D, Molofsky AB, Liang HE et al Eosinophils sustain adipose alternatively activated macrophages associated with glucose homeostasis. Science 2011;332:243–7. 21436399 10.1126/science.1201475 PMC3144160

[108] Yang Z, Grinchuk V, Smith A et al Parasitic nematode-induced modulation of body weight and associated metabolic dysfunction in mouse models of obesity. Infect Immun 2013;81:1905–14. 23509143 10.1128/IAI.00053-13 PMC3676010

[109] Berbudi A, Surendar J, Ajendra J et al Filarial infection or antigen administration improves glucose tolerance in diet-induced obese mice. J Innate Immun 2016;8:601–16. 27544668 10.1159/000448401 PMC6743339

[110] Chen Y, Lu J, Huang Y et al Association of previous schistosome infection with diabetes and metabolic syndrome: a cross-sectional study in rural China. J Clin Endocrinol Metab 2013;98:E283–7. 23275524 10.1210/jc.2012-2517

[111] Aravindhan V, Mohan V, Surendar J et al Decreased prevalence of lymphatic filariasis among diabetic subjects associated with a diminished pro-inflammatory cytokine response (CURES 83). PLoS Negl Trop Dis 2010;4:e707. 20559443 10.1371/journal.pntd.0000707 PMC2886036

[112] Hays R, Esterman A, Giacomin P et al Does *Strongyloides stercoralis* infection protect against type 2 diabetes in humans? Evidence from Australian Aboriginal adults. Diabetes Res Clin Pract 2015;107:355–61. 25656764 10.1016/j.diabres.2015.01.012

[113] Wiria AE, Hamid F, Wammes LJ et al Infection with soil-transmitted helminths is associated with increased insulin sensitivity. PLoS One 2015;10:e0127746. 26061042 10.1371/journal.pone.0127746 PMC4464734

[114] Wiria AE, Wammes LJ, Hamid F et al Relationship between carotid intima media thickness and helminth infections on Flores Island, Indonesia. PLoS One 2013;8:e54855. 23365679 10.1371/journal.pone.0054855 PMC3554693

[115] Hicks JH, Kremer M, Miguel E The case for mass treatment of intestinal helminths in endemic areas. PLoS Negl Trop Dis 2015;9:e0004214. 26492528 10.1371/journal.pntd.0004214 PMC4619642

